# Exploring the advances of biosensing technology for the detection of plant pathogens in sustainable agriculture

**DOI:** 10.3389/fbioe.2025.1674574

**Published:** 2026-01-12

**Authors:** Sneha Shikha, Saurabh Dubey, Ranveer Kumar, Banothu Chandrashekar, B. K. Namriboi, Bhagyashree Bhatt, Subhashish Sarkhel, Abhijeet Shankar Kashyap, Nazia Manzar, Abhijeet Ghatak

**Affiliations:** 1 Bihar Agricultural University, Sabour, Bihar, India; 2 Dr. Rajendra Prasad Central Agriculture University, Pusa, Bihar, India; 3 G.B. Pant University of Agriculture and Technology, Pantnagar, Uttrakhand, India; 4 ICAR-National Bureau of Agriculturally Important Microorganisms, Mau, Uttar Pradesh, India

**Keywords:** biosensors, plant disease detection, precision agriculture, nanotechnology, pathogen monitoring

## Abstract

Plant pathogens, including fungi, bacteria, viruses, and nematodes, remain major constraints to global agricultural productivity, threatening food security and ecosystem sustainability. Conventional diagnostic methods such as culture-based assays, ELISA, and PCR provide reliable results but are often time-consuming, resource-intensive, and limited in field applicability. Recent advances in biosensing technology have emerged as transformative alternatives, offering rapid, sensitive, and cost-effective detection of plant pathogens. Biosensors (integrating bioreceptors with electrochemical, optical, or piezoelectric transducers) enable real-time monitoring of pathogens at very low concentrations, often before visible symptoms appear. Innovations such as nanomaterial-enhanced platforms, CRISPR-based biosensors, microfluidics, and paper-based devices have improved detection accuracy, portability, and user-friendliness, making them suitable for field deployment. Furthermore, coupling biosensing with digital agriculture tools, artificial intelligence, and IoT facilitates predictive diagnostics, precision crop management, and environmentally sustainable practices. Despite these advances, challenges remain in ensuring long-term stability, affordability, and scalability, particularly for smallholder farmers. Addressing these gaps is essential to achieve widespread adoption. Overall, biosensing technologies hold significant potential to revolutionize plant disease management, minimize yield losses, reduce chemical dependency, and strengthen climate-resilient, sustainable agriculture.

## 1 Background

**FIGURE 1:**
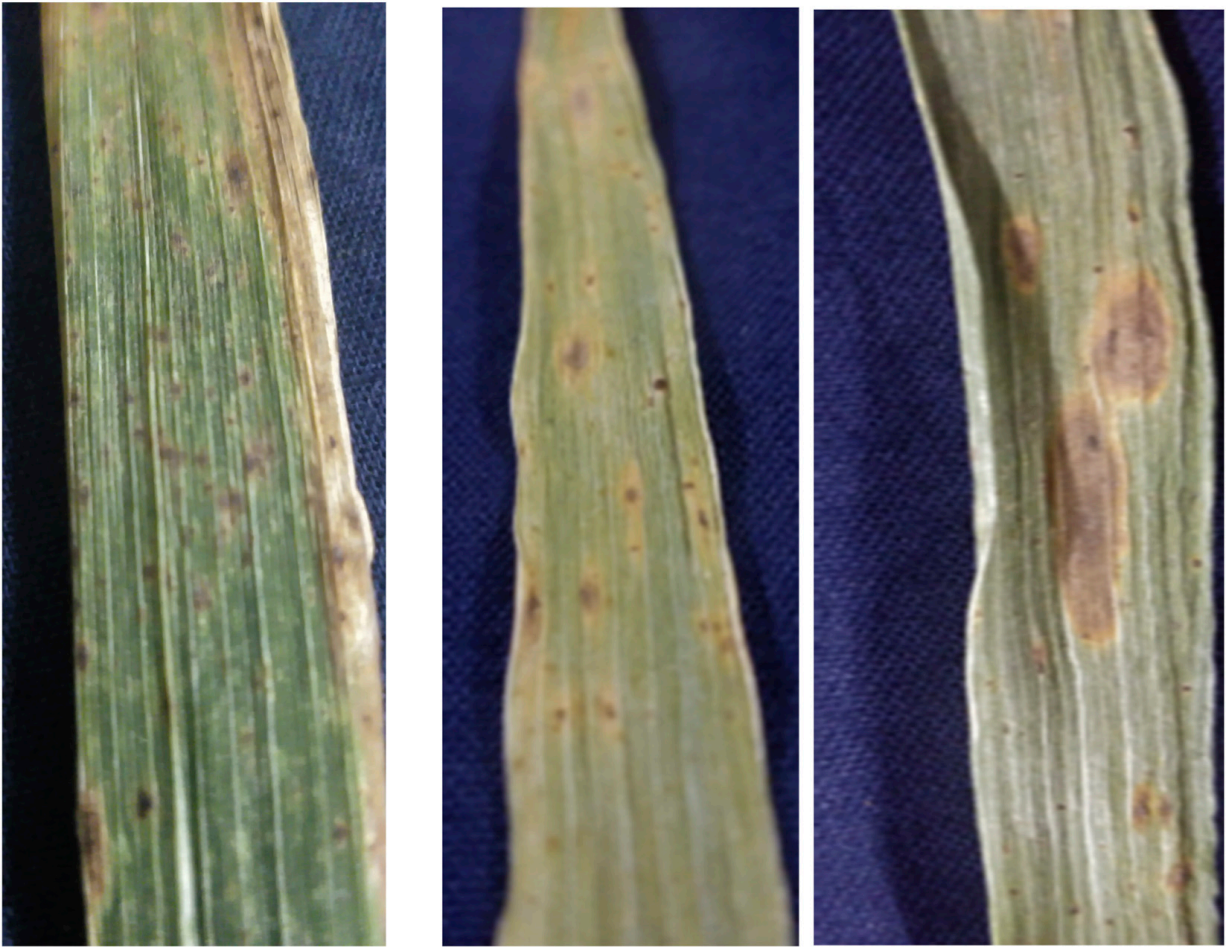
Symptoms of leaf blight of wheat.

Plant pathogens, such as fungi, bacteria, viruses, nematodes, and oomycetes, are a serious threat to agricultural productivity in the world (Kashyap et al., 2025). These pathogens result in a range of diseases, reducing yield, as well as impacting the quality and nutritional value of food. Interactions between plants and pathogens have developed over thousands of years, enabling plants to develop a plethora of defense mechanisms, while pathogens have evolved a multitude of strategies to evade plant defenses. Plant diseases create billions of dollars of economic losses each year. For instance, *Puccinia* spp. (wheat rust) and *Phytophthora infestans* (late blight in potatoes and tomatoes), have led to famines when originally introduced to new regions, and some still impose significant economic strain on agricultural production. *Xanthomonas* spp., and *Pseudomonas syringae*, are examples of bacterial pathogens that cause wilting, development of lesions, and overall plant death. Similarly, viral pathogens, such as Tobacco mosaic virus andTomato yellow leaf curl virus, disperse through the environment and can completely destroy fields with no significant chemical control. Nematodes affect root systems and prevent nutrient uptake, while oomycetes are likely to thrive in water-saturated environments, adding complexity to plant protection (Figure 1).

The use of high-density monocultures by modern agriculture increases susceptibility to pathogens. Homogeneous host environments allow for quick dispersal and adaptation of the pathogen and result in regular outbreaks. Global change also increases these risks by changing the pattern of temperatures, precipitation, and humidity, increasing the range of pathogen distributions to previously unaffected areas, and a new set of challenges to food security at the global level. Human activities, which involve the use of global exchange and plant movement, also spread diseases (Santini et al., 2018). The introduction of the fungus *Phytophthora ramorum*, which causes sudden oak death in Europe and the Nearctic region, illustrates the unforeseen consequences of the dispersal of a pathogen and the need for biosecurity protocols.

Plant diseases pose direct as well as indirect costs to agriculture. Smallholder farmers in developing areas may suffer economically due to the yield losses, and the costs of managing the disease (including chemical treatments, biological management, and resistant cultivars) may be prohibitive (Hussain, 2024). The excessive use of chemical pesticides has led to environmental degradation, resistance against pesticides,and also health hazards,and therefore sustainable disease management is required. Effective strategies have been developed as integrated disease management (IDM) strategies that combine practices of a given culture, resistant varieties, using chemicals and biological control, and sophisticated monitoring systems (Kashyap et al., 2025). However, the pathogens remain adaptive, thus remaining a challenge to control. In addition to economic consequences, plant pathogens have an impact on food security, nutritional value, and the sustainability of agroecosystems (McDonald and Stukenbrock, 2016). Crop failures can result in a food crisis, increase the market prices, and put the lives of millions of peoples at risk. Selective loss of the weak crop varieties also affects agricultural biodiversity and ecosystem resilience. Therefore, biology, epidemiology,and host-pathogen interactions of pathogens are to be perceived in order to generate early alert systems, predictive models, and new management solutions that do not undermine food production in regard to environmental sustainability.

The new technology has increased the research and management of pathogens. With the assistance of molecular techniques, such as polymerase chain reaction (PCR), next-generation sequencing (NGS), and metagenomics, the identification of the pathogen can be done at high levels even in limited populations (Kashyap et al., 2023; Manzar et al., 2022), and active intervention is possible. This is through remote sensing, geographic information systems (GIS),and decision support systems, which are digital tools used in agriculture that have made it possible to monitor and forecast the disease in real time. Such technologies are even enhancing the savvy of agriculture, particularly against climate change and the global population pressure. Pathogens tend to have complicated life cycles, host specificity, and environmental adaptability, which makes them difficult to detect and contain. Such fast evolution may lead to virulent strains that can overcome resistant cultivars or current treatment, which may result in the necessity to continue research on new methods of detection, sustainable methods of managing, and breeding of resistant strains. Disease management also demands collaboration between scientists, policymakers, farmers, and industry actors in order to maintain a timely implementation of strategies, dissemination of knowledge as well as adherence to regulations.

### 1.1 Global research trends in plant biosensors

The research on biosensors in plant pathogen detection has increased significantly over the last 5 years, as it demonstrates an increasing need for fast and sustainable means of diagnostics in agriculture. Research data across the literature shows that there is an obvious positive trend, and the number of papers is growing each year, as in 2019, its total was around 120 articles, whereas in 2023, it reached approximately 300 papers (Olson and Bae, 2019). This doubled increase highlights the increased appreciation of biosensing technologies as essential elements to sustainable crop protection methods. China, the United States, and India have published the largest number of publications, whereas European countries, such as Germany, the United Kingdom, and the Netherlands, have also proven to be very active in their research (Talwar et al., 2023). The major institutions like the Chinese Academy of Sciences, Wageningen University, the University of California system, and the Indian Council of Agricultural Research have become the key centers of research and development of fundamental research and applied applications in plant biosensing (Talwar et al., 2023) (Table 1).

**TABLE 1:** Leading countries and institutions contributing to plant biosensor research (2019–2023).

Rank	Country/Institution	Contribution (%)	Notable focus areas	References
1	China	28%	Nanomaterials, electrochemical biosensors	Narware et al. (2025)
2	United States	21%	CRISPR-based platforms, IoT integration	Chaturvedi et al., 2025; Deng et al., 2025
3	India	14%	Portable field biosensors, sustainable agriculture	Yadav and Yadav (2025)
4	Germany	8%	Microfluidics, lab-on-a-chip technologies	Narware et al. (2025)
5	Netherlands (Wageningen University)	7%	Precision agriculture, pathogen detection workflows	Narware et al., 2025; Chaturvedi et al., 2025
6	United Kingdom	6%	Optical biosensors, field validation studies	Narware et al. (2025)

Several trends can be identified according to thematic analysis with the help of keyword co-occurrence. Such keywords as biosensors, plant pathogen detection, nanotechnology, and sustainable agriculture can be seen on multiple occasions. It can be seen that there are three major research clusters, including (1) the use of nanomaterials to improve sensitivity and signal transduction (Narware et al., 2025), (2) the use of molecular biosensing platforms (CRISPR-Cas systems and nucleic acid-based sensors) to identify pathogens precisely (Chaturvedi et al., 2025), and (3) the creation of field-oriented technologies, such as portable paper-based technologies, Internet of Things (IoT) -enabled biosensors, and microfluidic devices (Yadav and Yadav, 2025). New research is more concerned with Artificial Intelligence–based diagnostics, on-demand testing, and real-time IoT integration, which means that lab-confined systems are being replaced by field-deployable biosensing systems (Deng et al., 2025).

In line with these findings, our analysis of citations underscores prominent and impactful publications in interdisciplinary journals, including Biosensors and Bioelectronics, Sensors, Analytical Chemistry, and Frontiers in Plant Science. The focus on nanomaterial-based biosensors and CRISPR-based platforms in this citation analysis shows their prominence and citation attention and reflects a significant development in agricultural biotechnology and applied diagnostics (Narware et al., 2025; Chaturvedi et al., 2025). Studies that explicitly link biosensing technologies to precision agriculture and sustainable crop management seem to garner more visibility and scientific impact, signifying the applicability of this area of research in agricultural and environmental applications (Laveglia et al., 2024).

Although there have been significant advances, there are still a lot of gaps to overcome. The majority of research still exists in a laboratory environment and has been limited in terms of validating in the field under a variety of conditions. Major challenges that have yet to be addressed include long-term stability and robustness of sensors during changing environmental conditions, as well as integrating biosensors into AI and IoT platforms for real-time disease monitoring (Deng et al., 2025). Furthermore, the economic viability, scalability, and adoption by smallholder farmers are still under-researched. The solution to these challenges allows the development of low-cost, strong, and digitally integrated biosensing technologies that can support plant health monitoring towards climate resilient and sustainable agriculture.

### 1.2 Plant pathogens in agriculture

Plant pathogens are diverse microorganisms and organisms that may cause diseases in crops and lead to severe decline in agricultural productivity, food security, and ecological balance (Manzar et al., 2024). To ensure sustainability in agriculture, it is vital to understand and control plant pathogens as plant disease leads to significant loss of crop yield and poor quality of crops. Plant pathogens do not necessarily affect their host plants in a uniform manner since they depend upon climatic factors, situations of agricultural practice, and host plant susceptibility (Barrett et al., 2009). Understanding the biology, diversity, and detection of plant pathogens is essential in devising sound management strategies that promote the safety of food systems globally.

Plant pathogens are generally separated into four groups: bacteria, viruses, fungi, and nematodes, with unique established mechanisms of infection and pathogenicity. Bacterial diseases in plants are predominantly associated with pathogenic species of *Xanthomonas* spp. and *Pseudomonas syringae*, which are known to infect a wide range of economically important crops. These bacteria infect plants through inherent openings or injuries, causing symptoms including leaf spots, blights, and wilts (Patel et al., 2025). Obligate intracellular parasites include viral pathogens such as Tobacco mosaic virus (TMV) and Cucumber mosaic virus (CMV),which suppress plant metabolism and growth and commonly enter the cell by using insect vectors or mechanical damage. Most plant diseases worldwide are caused by fungal pathogens such as *Fusarium* spp., *Alternaria* spp., and *Phytophthora* spp., which induce wilts, rots, and blights through spore germination, tissue invasion, and toxin production. (Doohan and Zhou, 2017). Microscopic parasitic worms known as nematodes, including *Meloidogyne* spp. and *Heterodera* spp. infect plant roots and, in addition to blocking nutrient absorption, predispose plants to secondary infection. These pathogens occur in great numbers within the plant kingdom and pose a major challenge in detection and control due to the diversity of pathogens and their complex life cycles, in addition to the biology and ecology of the pathogens, which are required to manage and control the losses*Xanthomonas* spp. and *Pseudomonas syringae*, which are responsible for leaf spots, blights, wilts, and cankers, are just a couple of examples. These pathogens normally prefer warm, humid environments and spread quickly, requiring early detection and manageme. Tobacco mosaic virus (TMV) and Cucumber mosaic virus (CMV) are well-known viruses. Viral pathogens disrupt plant metabolism, causing mosaic patterns, stunted growth, and deformed fruits. Unlike their bacterial counterparts, there are no chemical controls for management. Management relies on resistant varieties and vector control (Eisenreich et al., 2019).

*Fusarium* spp., *Alternaria* spp., and *Phytophthora* spp. cause wilts, rots, and blights. Fungi penetrate the tissues of plants and often produce enzymes and toxins, and their growth is influenced by moisture and temperature (Godswill Awuchi et al., 2022). Management tactics include the use of fungicides, resistant cultivars, and crop rotation.

Plants infected with nematodes show stunted growth and yellowing and often have decreased yields, but nematodes can also introduce secondary infections. Integrated approaches, including crop rotation, resistant varieties, and biological controls, are proven useful for control.

### 1.3 Economic and ecological impact

The economic impact that plant pathogen outbreaks cause is large and manifests in the reduction of crop yield, quality, and marketability. Worldwide estimates indicate that plant diseases account for a loss of approximately 10%–16% of annual agricultural production, which translates to billions of dollars in economic damage (Junaid and Gokce, 2024). More than the economic loss, the importance of the ecological consequences of plant pathogens cannot be overstated. Disease epidemics can disrupt agroecosystems, alter species composition, and impact soil health. Infected plants can serve importantly as reservoirs for pathogens, aiding in pathogen persistence across planting seasons (Ratnadass et al., 2012). Additionally, if left unmanaged, agroecosystems often resort to abundant use of chemical control measures that can have a negative impact on non-target organisms, pollinators, and soil microbial diversity, supplanting the need for an integrated, environmentally sustainable disease management strategy.

### 1.4 Current methods for detection

The ability to quickly and accurately identify plant pathogens is vital in order to effectively manage the disease and minimize yield losses. Traditional detection techniques include visual inspection, culture-based techniques, and serological techniques. Visual detection uses disease symptoms (i.e., leaf spots, chlorosis, or necrosis) as a means of identification. However, diagnostic accuracy may be limited by graded symptom similarity that exists with various diseases, as well as the delayed onset of symptom development (López et al., 2009). Culture techniques used for isolating the pathogen(s) on selective media, followed by morphological and biochemical analysis. While these methods provide reliable information, they are labor-intensive and also time-consuming. In serological techniques like enzyme-linked immunosorbent assay (ELISA) testing, specific antibodies are utilized in order to identify the pathogen by detecting pathogen antigens with moderate sensitivity and specificity. Modern molecular techniques, polymerase chain reaction (PCR), loop-mediated isothermal amplification (LAMP), and next-generation sequencing (NGS), facilitate the rapid, sensitive, and accurate detection of pathogens at the DNA or RNA level, even within asymptomatic host plants. These molecular techniques have greatly improved pathogen identification with high-throughput, real-time, and accurate laboratory diagnostics - crucial for a timely response and outbreak management (Okiro et al., 2019).

Although traditional detection methods are historically important, they have major drawbacks. For example, visual inspection and symptom-based diagnoses are subjective and unreliable, particularly in the early stages of infection. Culture-based methods are limited because some pathogens grow slowly and/or some are obligate parasites that cannot be cultured, as well as requiring specialized laboratory settings (Wang et al., 2022). Serological methods have limitations with cross-reactivity, sensitivity, and for low-titer infections. Given these limitations, it is imperative to incorporate modern biosensing and molecular diagnostic techniques into plant disease surveillance systems to improve early detection, minimize the amount of time spent working with labor-intensive methods, and further develop sustainable disease management practices that limit economic losses and ecological disruption (Wang et al., 2025).

## 2 Importance of early and accurate detection for sustainable agriculture

Sustainable agriculture has become an important global goal in the face of rising demands for food, increased populations, and growing environmental pressures. Plant health is also crucial in meeting agricultural sustainability because crop losses attributed to pathogens put food security and economic viability at risk. Plant diseases, which may be caused by a diverse group of bacteria, viruses, fungi, and nematodes, can affect crop yield and quality, resulting in detrimental consequences to both the farmer selling crops and the end consumer. Effective management of plant diseases is achieved through timely management strategies and accurate, rapid detection of targeted pathogens (Srivastava et al., 2025). Traditional techniques, visual observations, culture-based methods, and molecular assays are practiced widely, but are not well suited to provide adequate timely, real time targeted, on-site precise diagnosis. Delayed identification of the plant pathogen provides the opportunity for the pathogen to spread uncontrollably and increases pathogen management costs through higher agrochemical use, which conflicts with the principles of sustainable agriculture. Hence, accurate timely detection of pathogens is needed for practical reasons and to protect the environment, supporting intentional accurate,and efficacious targeted crop protection (Huded et al., 2023).

Biosensing technology has become a revolutionary method in the detection of plant pathogens, and it provides a blend of sensitivity, specificity, and fast response that is not always provided by the standard methods (Dyussembayev et al., 2021). Biosensors are analysis devices used to transform a biological response into a measurable signal, which allows detecting pathogens at extremely low concentrations, sometimes even before symptoms manifest themselves. The fundamental elements of a biosensor are usually a bioreceptor, which engages with the target pathogen, and a transducer, which generates the interaction into any of the following measurable signals: electrical, optical, or mechanical. Further improvements in biosensor performance occurred due to advances in nanotechnology, microfluidics, and molecular biology which have allowed the creation of portable, less expensive, and easy-to-use biosensors that can be used in the field (Srinivasan and Tung, 2015). The innovations can be applied especially to the agricultural setting, in which quick decision-making is necessary to avoid disease outbreaks and reduce losses. Biosensors have the potential to promote precise actions through targeted pesticide application, improved irrigation methods, and the use of resistant crop strains, thereby supporting the larger objectives of integrated pest management and sustainable agriculture.

Biosensing technology has a broader scope than pathogen detection and provides an important connection to more efficient use of resources and environmental stewardship (Kirsch et al., 2013). Most conventional strategies to manage disease include applying a chemical preventatively or indiscriminately, leading to environmental pollution, pathogen resistance, and negative effects on non-target organisms. Biosensors permit the real-time observation and accurate identification of pathogen and pest dynamics, which leads to precision agriculture that could limit excessive chemical use and advance environmentally sustainable practices (Pal et al., 2025). Additionally, by integrating biosensing with digital agricultural systems, like wireless data networks and geographic information systems or apps to smartphones, enables remote observation and data-driven decisions. Digital integration with biosensors also increases the ability to observe disease dynamics over a broad regional context and use that data to forecast disease or pest outbreaks and manage them preventively. This presents a format of crop protection that moves from a climate of reaction to a system of prevention, and hence, sustainability.

Recent research highlights a variety of biosensing platforms for detecting plant pathogens, including electrochemical, optical, piezoelectric, and microfluidic biosensors, each offering distinct advantages in terms of sensitivity, portability, and the ability to test for multiple pathogens at once (Liao et al., 2019). For instance, electrochemical biosensors provide rapid, quantitative measurements of pathogen levels, while optical sensors can detect pathogens non-destructively through fluorescence or color changes. Microfluidic biosensors are particularly effective for simultaneously testing small sample volumes for multiple pathogens, which is crucial for complex field conditions. These technologies are collectively transforming plant disease management, enabling faster, more precise, and sustainable agricultural practices (Khan and Munawar, 2023). However, challenges remain regarding standardization, long-term stability, in-field validation, and scalability. Overcoming these limitations is essential for these biosensing platforms to reach their full potential in agriculture.

### 2.1 Principles of biosensing technology

Biosensors are analytical instruments that use a biological recognition element combined with a physicochemical transducer and are designed to measure, detect, and quantify biological analytes (Grieshaber et al., 2008). They enable fast, sensitive, and selective determination or detection of target molecules, pathogens or environmental contaminants by converting biochemical response into a measurable signal. Each biosensor has at least three core components, the bioreceptor interacts with the analyte selectively, the transducer converts the biochemical interaction into a measurable signal, and the signal processor amplifies and processes the output signal for quantification or display. The bioreceptors could be either enzymes, antibodies, nucleic acids, or a whole cell, depending on the properties of the target molecule, and the transducer could be optical, electrochemical, piezoelectric or thermal, depending on the type of signal generated and where biosensor signals will be used (Nakamura, 2012). Overall, these three components elaborate how effective the biosensor will be in sensitivity, viability, and versatility for its specific applications in medical diagnostics, environmental approaches,and agricultural systems (Table 2).

**TABLE 2:** Principles of biosensing technology.

Principle	Mechanism	Key components	Applications in plant pathogen detection	References
Biorecognition	Specific interaction between bioreceptors (e.g., antibodies, nucleic acids, aptamers, enzymes) and target analytes (pathogen proteins, DNA, toxins)	Bioreceptors (antibodies, aptamers, DNA probes, enzymes)	Detecting viral DNA/RNA, bacterial toxins, fungal enzymes	Naresh and Lee, (2021)
Signal transduction	Conversion of biorecognition event into a measurable signal (optical, electrochemical, piezoelectric, or thermal)	Transducers (optical fibers, electrodes, quartz crystals)	Identifying pathogen presence via electrical or optical changes	Ramesh et al. (2022)
Amplification	Enhancing weak signals to increase sensitivity and detection limits	Nanomaterials (gold nanoparticles, quantum dots, graphene)	Ultra-sensitive detection of low pathogen loads in crops	Kashyap et al. (2016)
Detection and processing	Processing of amplified signals into readable digital outputs	Microprocessors, biosensor software, AI integration	Real-time monitoring of pathogen spread in fields	Huang et al. (2023)
Output/Display	Final step showing results in qualitative or quantitative form	Screen displays, portable readers, smartphone integration	On-site rapid testing for early disease management	Katey et al. (2023)

### 2.2 Types of biosensors

**FIGURE 2:**
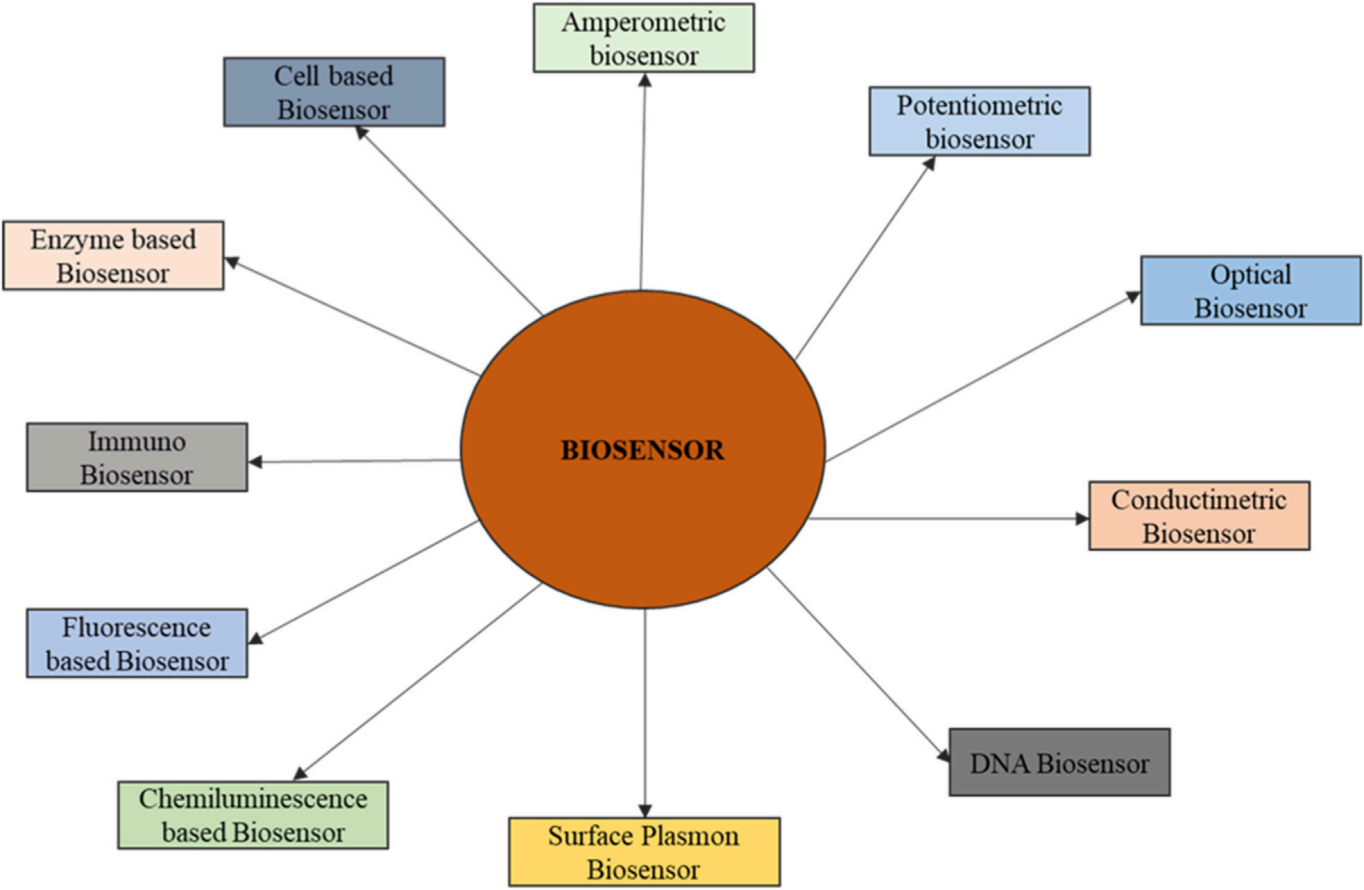
Types of biosensors.

Biosensors are categorized based on the type of transduction mechanism they employ (Figure 2) (Yang et al., 2020).

- Electrochemical Biosensors: These sensors detect electrical changes such as current, potential, or impedance upon analyte recognition. They are highly sensitive, low-cost, and compatible with miniaturization, making them ideal for portable devices (Brett, C. 2022).
- Optical Biosensors: Based on the detection of light changes, these sensors use principles like absorbance, fluorescence, luminescence, or surface plasmon resonance. They allow real-time and label-free detection, which is advantageous for continuous monitoring.
- Piezoelectric Biosensors: Piezoelectric biosensors use measurements of mass change that occur on the surface of piezoelectric crystals. When an analyte binds or attaches to the sensor surface, the binding produces a detectable frequency shift, so piezoelectric biosensors are very useful for monitoring molecular interactions.
- Immunosensors: Immunosensors, in contrast, are a distinct category of biosensors that derive their high specificity from antigen–antibody interactions. Immunosensors can be coupled to electrochemical or optical platforms, which enhance their limits of detection substantially.

**FIGURE 3:**
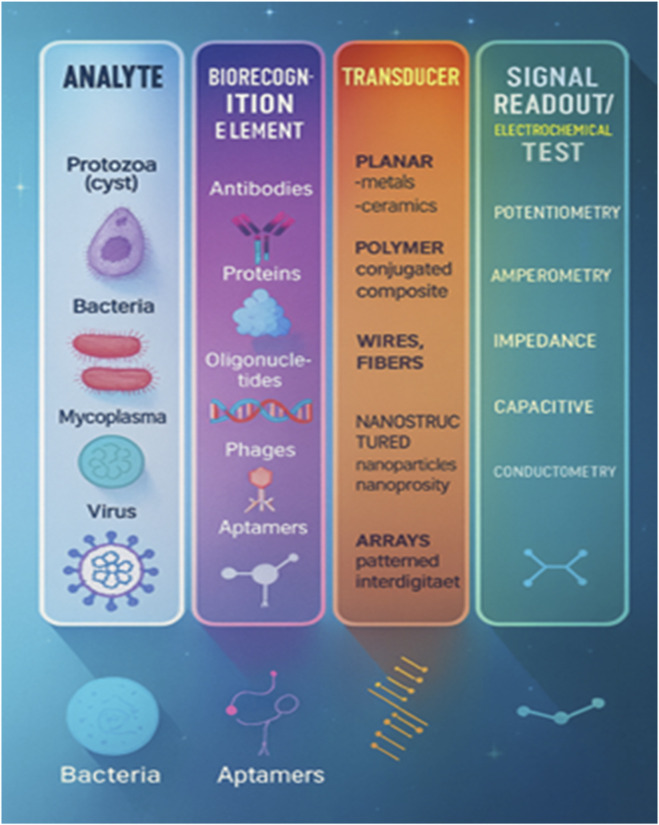
Different types of electrochemical sensors and analyte interaction with different biorecognition elements.

Ultimately, the selection of a biosensor type and category depends on the intended application, the required level of sensitivity, and the environmental conditions, enabling researchers to choose the most suitable option for their study (Figure 2; Figure 3).

### 2.3 Key performance metrics

Biosensor performance is evaluated via performance attributes that are critical: sensitivity, specificity, limit of detection (LOD), and response time (Johnson and Mutharasan, 2014). Sensitivity, the ability of the sensor to generate a detectable output that correlates with analyte concentration, is a significant metric to allow for the detection of trace pathogens. Specificity allows the biosensor to distinguish the analyte of interest from neighboring or interfering structural entities, enabling accurate detection. The LOD represents the concentration of an analyte that can be detected and reproducible, and is important for early detection of the presence of pathogens. The response time of the biosensor describes the time the sensor produces a detectable output once exposed to the analyte, which influences whether the biosensor can be used in real-time detection. In addition to these performance metrics, some factors that also affect biosensor performance include stability, reproducibility, and the lifespan of the biosensor system (Demir et al., 2024). Often, an optimal level of performance in each of these attributes is related to the use of combinations of bioreceptor design, transducer selection and surface chemistry, often supported by computational modeling and advances in materials science.

## 3 Advances in biosensing for plant pathogen detection

Effective detection and monitoring of plant pathogens are critical for the maintenance of plant health, and for strengthening our global food security, as pathogens such as bacteria, viruses, and fungi are believed to be major contributors to agricultural losses (Strange and Scott, 2005). Conventional diagnostic protocols, such as microbiological culture, polymerase chain reaction (PCR), and enzyme-linked immunosorbent assay (ELISA), have the benefit of reliability and accuracy. However, their use is limited by being labor-intensive, requiring specialized laboratory protocols, and trained personnel, thus limiting application to diagnostic methods that are very rapid and performed in the field. With new developments in biosensing technology, rapid, highly sensitive, and field-based alternatives are now being viewed as potential tools for pathogen detection (Patel et al., 2022).

### 3.1 Nanomaterial-based biosensors

Biosensors based on nanomaterials are of great interest because of the unique physicochemical properties of materials at the nanoscale that greatly enhance detection performance. Nanoparticles such as gold, silver, and carbon-based materials (carbon nanotubes, graphene, quantum dots) serve as functional scaffolds to attach biorecognition elements (antibodies, aptamers, nucleic acid probes) (Fritea et al., 2021). The very high surface area-to-volume ratio, along with their unique optical, electronic and catalytic properties, allows these nanoparticles to effectively capture pathogens and amplify the ensuing signals. For example, gold nanoparticles can and regularly are used in colorimetric assays, where they change color upon aggregation with pathogens. Silver nanoparticles, in surface-enhanced Raman scattering (SERS) biosensors, can detect very low concentrations of pathogens, and carbon nanomaterials can improve electrochemical biosensors due to enhanced electrical conductivity to detect pathogens such as Xanthomonas spp. and tobacco mosaic virus (TMV) (Khan et al., 2025). Additionally, the speed, potential for miniaturization, and style of device portability complemented by apps means these biosensors have high applicability for real-time monitoring of plant health due to the ability to integrate with smartphone or wireless devices to enable rapid response and timely response to disease (Lan et al., 2017).

### 3.2 DNA/RNA-based biosensors (Genosensors)

Two types of innovative technologies used for pathogen detection that focus on targeting nucleic acids are genosensors and CRISPR-based biosensors. Genosensors utilize complementary DNA or RNA probes that hybridize to the nucleic acids of a pathogen. The resulting signal is then measured, including through electrochemical, optical or piezoelectric methods (Mobed et al., 2019). Nucleic acid amplification methods, such as PCR or LAMP can be used in these platforms to improve sensitivity. Similarly, CRISPR-based biosensors detect the target through sequence-specific nuclease activity CRISPR-associated proteins like Cas12, or Cas13, and once target binding occurs, the proteins undergo collateral-cleavage of a reporter molecule and a detectable signal is generated. Importantly, both biosensor technologies can be designed as multiplexed assays to detect several pathogens at once, which enhances their potential for diagnostic utility, particularly in agriculture (Hong, 2019).

Genosensors have been successfully applied to detect numerous plant pathogens, such as *Xanthomonas citri* subsp. *citri* (synonym *X. axonopodis* pv. *citri).* in citrus, *Phytophthora infestans* in potatoes, tobacco mosaic virus (TMV), cucumber mosaic virus (CMV), *Pseudomonas syringae*, and *Erwinia amylovora*. While high-accuracy, laboratory-based systems are reliable, they are often expensive and require skilled personnel. In contrast, portable genosensors are being developed for rapid, user-friendly field detection, though they might offer slightly lower sensitivity. Recent advancements in microfluidics, paper-based devices, and CRISPR-based devices are addressing the limitations of current genosensors, providing practical and scalable solutions for managing crop diseases (Zhao et al., 2024).

### 3.3 Immunosensors (Antibody-based detection)

Immunosensors represent an advanced type of biosensor that exploits the highly specific nature of antigen-antibody interactions and has been used to detect pathogens quantitatively (Leva-Bueno et al., 2020). The principle of an immunosensor arises from the selective binding of an antibody immobilized on the transducer’s surface with its specific antigen that is in the assay sample. After the binding event, the physicochemical reaction of binding causes a measurable response, each of which is eventually transduced into a detectable signal (electric signal, optical signal, or change in mass of object). Because of the specific nature of the binding proteins used, this type of biosensor is able to detect target pathogens despite complex background matrices, meaning early detection and specificity (Singh et al., 2016). Immunosensors show potential to effectively detect both bacterial and viral pathogens. For bacterial detection, immunosensors allow for the rapid identification of a broad spectrum of pathogens associated with humans and plants, thereby enabling action to be taken quickly for the appropriate disease. Immunosensors are also being increasingly used in virology, even with emerging viral pathogens, as traditional culture methods are impractical due to their laborious and slow nature of the technique. Immunosensors can also provide real-time or near real-time results, which is a clear advantage over traditional diagnostics, such as ELISA, or PCR, that require significant method development, sample preparation, or both using sophisticated laboratory high-tech approaches (Castillo-Henríquez et al., 2020).

The performance of immunosensors is typically gauged with respect to sensitivity, specificity, and multiplexing capability (Munge et al., 2016). Sensitivity indicates the sensor’s ability to detect very little native antigen concentration (potentially at femtomolar or picomolar concentrations), and is determined by the affinity of the antibody and how efficient the transducer is. Specificity refers to the sensor’s ability to reduce cross-reactivity of non-target molecules, which is important in more complex settings where multiple microbial species are present. Advances in microfabrication methods and novel nanomaterials have enabled multiplexing that gives the ability to detect multiple pathogens at once within a single assay system. This characteristic is invaluable in agricultural and clinical settings, where rapid screening of multiple threats is of primary concern. Antibody based immunosensors are an adaptable and very robust platform for a biosensing approach in today’s world and allowing it to be interfaced into a portable, user-friendly device that becomes significant for viable monitoring of pathogens *in situ*, essentially closing the feedback loop between laboratory-based diagnostic capabilities and actual viable applications in the field. Advancements in antibody engineering, surface chemistry and optimized transducer performancewill extend sensitivity, specificity and multiplexing capabilities further as immuno-sensor technology becomes an integral tool for pathogen detection.

## 4 Implementation and field deployment of plant biosensing technologies

### 4.1 Laboratory-based detection

Plant biosensors that are based in laboratories have played important roles in advancing the study of plant-pathogen interactions and in early detection of infections. Laboratory-based biosensing platforms employ techniques such as polymerase chain reaction (PCR), enzyme-linked immunosorbent assays (ELISA), and fluorescence- or electrochemical-based biosensing methods. Laboratory-based systems allow for the detection of pathogens in a controlled environment that provides high sensitivity, reproducibility, and accuracy, even at very low concentrations (Narware et al., 2025; Chaturvedi et al., 2025). Early detection of infections is paramount for prompt disease management, sometimes prior to the manifestation of any visible symptom. However, the complexity of these methods, reliance on advanced instrumentation and a skilled operator greatly restricts the utility of these methods to the laboratory setting, limiting their application to field conditions (Deng et al., 2025).

### 4.2 Field deployment of biosensors

Translating biosensors to agricultural fields presents unique challenges due to environmental variability. Factors such as temperature fluctuations, humidity changes, soil composition, and plant tissue characteristics can affect sensor sensitivity, reliability, and stability. Recent advancements in nanotechnology, microfluidics, and paper-based biosensors have facilitated the development of portable devices capable of real-time pathogen monitoring (Yadav and Yadav, 2025). Such devices are designed to be cost-effective, user-friendly, and robust, enabling farmers and field workers to perform rapid on-site detection, thereby enhancing the practical utility of plant biosensors in precision agriculture.

### 4.3 Challenges in translating laboratory prototypes to field use

Bridging the gap between laboratory prototypes and field-ready biosensors remains a major challenge. Maintaining accuracy and specificity under variable field conditions is difficult, as co-existing pathogens, environmental contaminants, and variations in plant tissue composition can interfere with detection signals. Integration with digital agriculture tools (such as IoT-based monitoring systems, smartphone interfaces, and geospatial mapping) offers potential to enhance real-time surveillance and support precision crop management (Deng et al., 2025; Chaturvedi et al., 2025). Nonetheless, standardization, long-term stability, reproducibility, and cost reduction are critical research priorities to ensure widespread adoption and scalability of field-deployable plant biosensing technologies.

### 4.4 Integration into sustainable agriculture practices

Effective field-deployable biosensors have the potential to revolutionize crop management. Early, accurate detection of pathogens can minimize yield losses, reduce unnecessary pesticide use, and support sustainable agricultural practices, thereby contributing significantly to global food security (Chaturvedi et al., 2025).

The convergence of biosensing technologies with sustainable agricultural practices has become a game-changing avenue in contemporary crop management. These innovative systems support the monitoring of plant health in real time and the detection of pathogens at their earliest stages, which is critical for preventing devastating losses in crop yield (Sarabandi et al., 2025). Biosensors provide reliable and on-time information about disease incidence, allowing farmers to apply targeted management practices that are specific to crop and field, and reduce the blanket application of pesticides. The precision approach improves crop yield while lowering environmental externalities associated with agricultural production. Recently, the incorporation of biosensing platforms into early warning systems has pushed proactive crop management even further, notifying farmers of threats, whether bacterial, viral or fungal, even before symptoms appear (Miguel-Rojas and Pérez-de-Luque, 2023). In this way, early warning systems improve decision making to better time irrigation, fertilization and disease management. By utilizing data in real time as conditions change, resource use improves, waste is reduced, and the economic damage associated with traditional recourse-based farming can be avoided.

In addition, monitoring via biosensors is vital for encouraging environmentally sustainable agriculture. These innovations enhance soil health and protect beneficial organisms by reducing the non-selective use of pesticides and fertilizers and limiting the chemical contamination of surface and groundwater (Chaturvedi et al., 2025). The integration of biosensing and digital agriculture, and smart farming platforms, opens the door for a much larger scale of monitoring and anticipatory analyses to improve sustainable crop production. The adoption of biosensing technologies within agriculture is an important step toward ensuring food security while supporting ecological integrity and improving resilience to plant diseases.

### 4.5 Economic considerations and scalability

The broad application of biosensing technologies in agriculture is closely associated with economic factors, such as sensor costs, maintenance and management, and personnel training. The high-precision biosensors often incorporate expensive materials and complex fabrication, which add significant expenses upfront. Besides initial expenses, recurring maintenance (calibration and consumable replenishment as well as software updates) presents a financial strain, especially to small-scale farmers (Becerra-Encinales et al., 2024). The deployment process also needs properly trained personnel who can use the sensors, analyze the data, and solve technical problems. Although necessary, the training programs require more time and money and might not be adopted in resource-limited environments.

### 4.6 Barriers to adoption in developing countries

Developing countries especially have economic and infrastructural constraints where technology, consistent electricity, and digital literacy are not always available. Small scale farmers find it difficult to afford more high-end biosensing equipment and unless the support is provided, they can easily end up underutilizing such technologies. Government subsidies, public-private partnership and cost-sharing models are some of the strategies that may be used to overcome other financial issues, but to achieve success in the adoption the strategy needs to be contextually adjusted and capacity building is needed to make users use and maintain biosensors (Jakku and Thorburn, 2010).

### 4.7 Scalability for large-scale agriculture

Scalability is a major issue that needs consideration when incorporating biosensing to large-scale agricultural activities. Although pilot studies and greenhouse trials are encouraging, Deployment on the field level requires standard protocols, robust data management and networked monitoring systems that are capable of processing significant amounts of information. Modular and portable biosensors have the potential to be expanded gradually, with little risk involved, and a wide range of coverage. It can be integrated with digital agriculture tools, such as Internet of Things and precision farming systems, which further increase scalability by enabling real-time monitoring of multiple sites and providing an opportunity to optimize resources and make decisions based on the data (Sharma and Shivandu, 2024). Consequently, economic feasibility and scalability should be addressed as one in order to achieve the potential of biosensing technologies in sustainable agriculture.

## 5 Challenges and limitations

While biosensing technology for plant pathogen detection holds great potential, there are significant challenges preventing its uptake as a sustainable agricultural strategy. A primary obstacle is sensor stability and durability in field conditions where temperature, humidity, soil characteristics and other factors will fluctuate and can impact sensor performance by generating inconsistent or inaccurate readings. Ensuring reliable operation in actual agricultural scenarios is a significant technological hurdle. Moreover, technical issues of cross-reactivity and specificity can lessen biosensor effectiveness, potentially leading to false positives or the inability to distinguish among closely related pathogens. In order to address these limits, highly selective bioreceptors and improved transduction mechanisms will need to be developed. Also, regulatory and standards issues present challenges besides the technical issues. Lack of transparency in approval processes and guidelines for marketing biosensing technology both impede commercialization and restrict farmer access to these technologies, particularly in countries with strict agricultural and food safety regulations, such as Canada. Economic costs and access can limit use as well, as pricing and technical expertise required for commercialization can limit access for small-scale or resource-poor farmers. To overcome these obstacles, biosensing technologies will need to be developed as cost-effective and user-friendly devices, along with training and support systems targeting access issues, in order to promote acceptable and equitable access and support effective and sustainable plant disease management in agricultural systems.

### 5.1 Future perspectives

Rising trends in the design of biosensors, including multiplexed detection, signal enhancement using nanomaterials, and artificial intelligence, are broadening the limits and effectiveness of plant pathogen diagnostics. There are prospects of commercialization and mass adoption, especially in precision and climate-resilient agriculture, where continuous monitoring is necessary in reducing the effects of biotic stressors. These technologies are potentially very important in changing the management of plant diseases, minimizing crop losses, as well as sustainability of agricultural activities in a global environmental problem.

## 6 Conclusion

The development of biosensing technologies is revolutionizing the process of detecting plant pathogens because it has introduced rapid, sensitive, and field-deployable solutions, which are in line with the principles of sustainable agriculture. CRISPR-based platforms, nanomaterials, and portable microfluidic systems can enable the biosensors to provide early and accurate diagnosis, which would reduce the use of chemical pesticides and minimize the losses of crops. The fact that they are compatible with digital and precision farming platforms also boosts real-time monitoring, resource efficacy, and preemptive disease control. Although there are difficulties in the area of scalability, sensor stability, and cost-effectiveness, the gap between laboratory prototypes and field applications can be closed with the help of constant innovations and appropriate policy frameworks. The application of biosensors to agricultural systems not only enhances food security but also fosters the ecological balance and climate resilience to climate stressors. Finally, biosensing technologies are an important way to reach sustainable crop protection and a better climate-smart agriculture.
